# Electroconvulsive therapy for refractory neuropsychiatric symptoms in frontotemporal dementia: a case report and literature review

**DOI:** 10.1192/j.eurpsy.2026.11751

**Published:** 2026-07-31

**Authors:** S. Orrego Molina, M. Sava Crihana, P. Nava García, M. E. Arévalo Gil, F. Pascual Pinazo, M. Sobredo Vega, M. J. Sánchez Artero, M. Nieves Carnicer

**Affiliations:** Psychiatry, Hospital Universitario Infanta Leonor, Madrid, Spain

## Abstract

**Introduction:**

Neuropsychiatric symptoms are common in dementia and contribute significantly to functional decline, institutionalisation, and caregiver burden. Symptoms such as depression, apathy, and oppositional behaviour can greatly affect quality of life and clinical outcomes. Electroconvulsive therapy (ECT), traditionally used for severe mood disorders, has emerged as a potential option for managing refractory behavioural and affective symptoms in dementia, especially when medications fail or are poorly tolerated.

**Objectives:**

To describe a clinical case of an elderly patient with frontotemporal dementia and severe food refusal who was successfully treated with ECT, and to discuss the therapeutic implications supported by a literature review.

**Methods:**

Case report and brief literature review on the use of ECT for neuropsychiatric symptoms in dementia.

**Results:**

An 87-year-old woman with a history of frontotemporal dementia was admitted to an internal medicine ward following a fall that resulted in cranioencephalic trauma. A psychiatric consultation was requested due to persistent refusal to engage in rehabilitation and maintain adequate oral intake. On initial assessment, the patient presented with a depressed mood and oppositional behavior. Antidepressant therapy was initiated but subsequently refused by the patient. Given the progressive deterioration of her nutritional status and continued resistance to treatment, electroconvulsive therapy (ECT) was proposed as a therapeutic intervention. The patient underwent nine sessions of bifrontotemporal ECT, which were well tolerated. Gradual clinical improvement was observed, including restoration of oral intake, improvement in mood, and increased cooperation with care. No significant cognitive or somatic adverse effects were noted. The patient was discharged with sustained clinical improvement.

**Conclusions:**

ECT appears to be a safe and effective therapeutic alternative for the management of refractory neuropsychiatric symptoms in patients with dementia, particularly in cases where pharmacological treatment is contraindicated, poorly tolerated, or ineffective, and where functional or nutritional decline poses additional clinical risks. Although clinical outcomes in this and other reports are encouraging, the current evidence base remains limited. Further well-designed studies are essential to establish robust and high-quality evidence supporting the use of ECT in this population. ECT should be integrated within an interdisciplinary therapeutic framework, ensuring close cognitive and somatic monitoring to optimise safety and efficacy.

**Disclosure of Interest:**

None Declared

